# Sleeping on Duty

**Published:** 1901-06-15

**Authors:** 


### SLEEPING ON DUTY.

War is war. Military law must, we suppose, be harsh and unrelenting, and when the success of great military operations and the lives of soldiers are at stake, the slightest breach of discipline must be severely punished. All this we grant. Yet we cannot but feel a sense of distress at the thought of soldiers, good men who have gone out with high hopes and have run their chance with others of dying for their country, ending their career, not with medals and handshakings, and a glorious welcome home, but as convicts undergoing penal servitude for sleeping while on duty. In England when a man sleeps on duty the chances are that his crime is the result, more or less immediate, of indulgence in alcohol. But in South Africa those actively engaged before the enemy have been practical teetotalers, and when these men have gone to sleep the probabilities are that their crime has been the outcome of circumstances over which they have had no control. Sleep is a curious physiological incident. Certain tissues in the body probably never sleep, but in most organs periods of activity alternate with periods of repose, and in all the higher animals the central nervous system at least once in the twenty-four hours enters into that condition of abeyance of its functions which we call sleep. What is the cause of this condition, the immediate mechanism of its production, we do not know. Some have imagined that during waking hours a gradual accumulation of some unknown toxin occurs which at last overcomes the brain, leading to sleep as surely and much in the same way as a dose of morphia; others that during our waking hours we use up something that is essential to full consciousness, and that at last a time arrives when the brain drifts into unconsciousness until this hypothetical substance is replaced; others that the neurons contract, and thus become isolated from one another; while others again believe exactly the opposite, namely, that the said neurons becoming relaxed and elongated lie in contact with each other, and thus short-circuit the whole nervous mechanism. All these are mere theories. The practical fact is that, putting on one side all question of poisoning by alcohol or other narcotic, sleep is induced on the one hand by fatigue to which it is a natural reaction, and on the other by absence of sensory stimuli; and also that when, as a result of fatigue, drowsiness overtakes the higher cerebral centres—the compelling mechanism in response to which all bodily activities take place—there is nothing left to drive the organism on to wakefulness, and, in the absence of external stimuli, sleep cannot be resisted. Many a drowsy soldier doing "sentry go" is saved from punishment by the wakefulness induced even by his monotonous exercise, and we may well suspect that many a soldier who in South Africa has gone to sleep on duty has done so partly as the natural consequence of fatigue and heat, and largely as the result of the absence of the tramp, tramp, by aid of which the sentry in Pall Mall is able to keep awake. The conditions in which outpost work has to be conducted in the presence of an enemy who would make a ready target of an upright sentry are conditions of physical restfulness; and in the presence of bodily fatigue, and the absence of mental or objective stimuli, we are not surprised that an undue proportion of those engaged in this work were overcome by their natural, and be it said healthy, tendency to sleep. Indeed, what is clear to us, as a mere problem in physiology, is that under such conditions sleep must often have had the absolute mastery. Doubtless the military authorities have considered all this, and, of course, Mr. Brodrick was correct in stating that according to military law this is a crime which subjects the offender to death (he stated in Parliament last week that two men in South Africa had actually been sentenced to death for this offence, but that their punishment had been commuted by Lord Kitchener to penal servitude); yet, knowing what we do concerning the impossibility of resisting sleep when it falls upon the higher centres, we cannot but feel a deep sorrow for men who, perhaps as a natural result of their own healthiness and bodily vigour, perhaps as a reaction to fatigue imposed upon them in obedience to orders, fell asleep while on duty, and ended their military careers in penal servitude.

